# Extensive Idiopathic External Root Resorption in First Maxillary Molar: A Case Report

**Published:** 2013-05-01

**Authors:** Behnam Bolhari, Naghmeh Meraji, Ali Nosrat

**Affiliations:** 1 Department of Endodontics, Dental School, Tehran University of Medical Sciences, Tehran, Iran; 2 Iranian Center for Endodontic Research, Research Institute of Dental Sciences, Shahid Beheshti University of Medical Sciences, Tehran, Iran

**Keywords:** Cone Beam Computed Tomography, Molar, Root Resorption, Tooth

## Abstract

External root resorption of permanent teeth is a multifactorial process. Several etiologic and predisposing factors have been related to external root resorption. Idiopathic external root resorption is defined as cases of external root resorption without a distinct etiologic factor. This article presents an extensive idiopathic external root resorption of maxillary first molar with irreversible pulpitis in an 18-year-old patient. The resorption was diagnosed in conventional radiographs and confirmed with Cone Beam Computed Tomography (CBCT) images. Unlike other reports in this field, and despite the severe resorption of all roots, there was no abnormal tooth mobility. Cold and electric pulp tests confirmed tooth vitality and revealed irreversible pulpitis. Therefore the exact etiology of the resorption remained unclear. Considering the poor prognosis due to severe root resorption, extraction and implant replacement was indicated.

## 1. Introduction

There are two types of external root resorption: physiologic and pathologic (1). Physiologic root resorption is associated with primary teeth and is a desirable procedure, because it results in exfoliation of the teeth, thereby allowing eruption of the permanent successors (1). Pathological root resorption is related to several local and systemic factors. Orthodontic therapy, trauma, periapical or periodontal inflammation, tumors, cysts, occlusal stress, impacted and supernumerary teeth, transplantation and re-implantation are among the local causes that could lead to pathological root resorption (2, 3). Endocrine imbalances, Paget’s disease of the bone, renal and hepatic disease have been reported as systemic causes of pathological root resorption (3).

Belanger and Coke (4) defined the term “idiopathic root resorption” as cases of root resorption where an etiological factor cannot be found. Idiopathic external root resorption can be either localized to the apical region or the cervical part of the root (5). The apical type causes a gradual shortening and rounding of tooth root (2). Patients with idiopathic root resorption are commonly asymptomatic clinically with an occasional complaint of tooth mobility. Therefore, the condition is usually found in routine radiographic examinations after a considerable amount of root structure is lost (6).

This article describes a case of extensive idiopathic external root resorption without bone resorption and mobility in first maxillary molar with irreversible pulpitis.

## 2. Case Report

An 18-year-old healthy male with a chief complaint of spontaneous pain and pain to cold and heat in the left posterior maxilla was referred to the Endodontic Department, Dental School, Tehran University of Medical Sciences. Medical history of the patient was non-contributory and there was no history of dental trauma.

In clinical examinations, left maxillary first molar had extensive coronal caries and a pulp polyp. The tooth was not sensitive to percussion and palpation. In periodontal examinations the probing depths were normal (<3 mm), with normal mobility. While the adjacent teeth responded normally, the first molar showed a prolonged response to cold test (Roeko Endo-Frost, Coltene Whaledent, Germany) and a very sharp response to electric pulp test (Parkell Inc., Farmingdale, NY, USA) in lower degrees.

**Figure 1.**
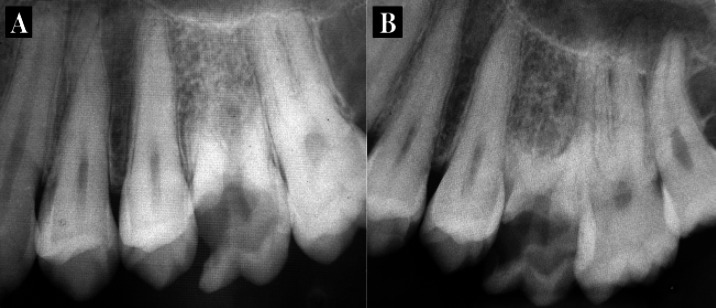
A) Periapical image of left maxillary first molar showing extensive coronal caries and severe root resorption in all three roots; B) A distal angulation showing extensive coronal caries and severe root resorption in all three roots

**Figure 2.**
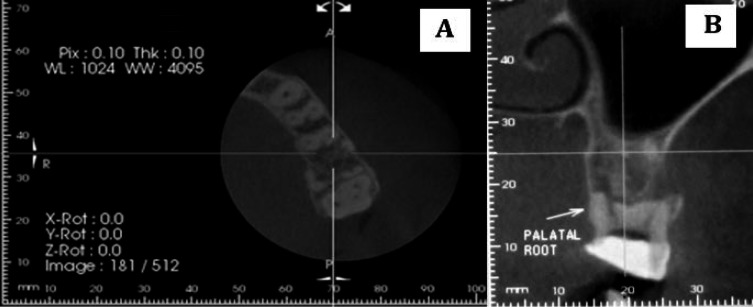
A) CBCT image of left maxillary first molar showing axial section in the mid-root. It shows extensive roots resorption; B) CBCT image of the tooth showing coronal view. Note the absence of roots due to extensive root resorption and normal bone structure

In radiographic examinations, extensive external root resorption was seen in all roots (Figures 1A, B). Considering the lingering pain and patient’s complaint, an emergency pulpotomy was performed on the first molar for immediate pain control. Since the whole of the palatal root could not be seen in periapical radiographs taken with different angulations, cone beam computed tomography (CBCT) images were obtained. They confirmed the presence of the extensive external root resorption in all roots with no periradicular osseous lesion (Figures 2A, B). Hematological investigations, including complete blood count, serum calcium, phosphorus, parathormone and alkaline phosphates levels were taken to exclude any systemic disorders. All were found to be within normal limits.

A pulpal diagnosis of irreversible pulpitis with pulp polyp was made for the tooth. Since the palatal root, which is required for dowel crown restoration, showed extensive resorption the prognosis for tooth retention was poor. Therefore, extraction and implant replacement was indicated.

## 3. Discussion

Root resorption in the permanent dentition has a pathologic basis, and is classified into two types of internal and external resorption. Internal resorption is relatively rare and occurs as a result of trauma or caries-related inflammation of the pulp. External root resorption has various causes, including: infection and inflammation, traumatic injuries, pressure/ mechanical stimulations, neoplastic conditions, systemic disorders, and idiopathic (3).

Conventional radiographic images are frequently used to detect root resorptions. However, the probability of false-negative results is a limitation of this method. Estrela et al. showed that CBCT scans were better for detecting root resorption than periapical radiographs; CBCT provided more accurate diagnosis with high-resolution images and lower observer interferences (7).

Treatment of inflammatory root resorption due to pulpal infection consists of long term calcium hydroxide therapy (8, 9). Other treatment plans such as application of low power lasers (10) or even single visit root canal therapy with gutta-percha (11) have also been suggested. The conventional treatment plan in invasive cervical root resorption is to expose the resorption lacuna orthodontically or surgically, remove the granulation tissue, and restore the resorptive defect (8). The main goal in these treatment plans is to remove the etiologic factor and stop the resorption process. However, in idiopathic resorptions the cause is unknown and therefore, the treatment is still a challenge.

External inflammatory root resorption involves the root and adjacent alveolar bone of a necrotic tooth (12). In the presented case, no alveolar bone resorption was seen and the tooth was not necrotic. Therefore we could not classify it as an external inflammatory root resorption.

Idiopathic root resorption was first defined by Belanger and Coke (4). This term is applied to cases of root resorption with unknown etiology. This type of resorption is infrequent and may appear in localized or multiple forms (13). The localized form occurs in one to three posterior teeth, whereas the multiple form may begin in the molars and bicuspids and eventually involve most of the dentition in a symmetrical pattern (2, 13).

Majority of the reported cases of “idiopathic root resorption” involve the apical part of several teeth and affects young people. The average reported age was 23.2 years (14). The teeth were vital, asymptomatic, responded positively to pulp tests, had normal probing depths, had intact crowns, and over time, became mobile without other clinical symptoms (13-18). Most of the teeth had attachment loss and insufficient alveolar bone support (19). Saravia et al. reported a case of multiple idiopathic root resorption in monozygotic twins in which the involved teeth had normal mobility(18). The radiographs revealed an almost identical resorptive pattern in both patients. This resorption involved all maxillary premolars and first molars and all mandibular premolars and molars.

Idiopathic apical root resorption does not seem to be mediated through pulp space. It is suspected that triggering factors exist for osteoblastic and odontoblastic activity causes root resorption. Therefore, treatment methods to arrest this type of apical resorption by interceptive endodontic procedures including pulp removal and placement of calcium hydroxide or any other intracanal medicament; obturation is not indicated (17).

In the presented case, there was only one involved tooth which had had symptomatic irreversible pulpitis, with prolonged response to the pulpal tests. There was no alveolar bone resorption, and the mobility of the tooth was normal. The extensive caries may be the cause of the atypical response of resorption, therefore this idiopathic root resorption is different from other reported cases.

Activation of interleukin (IL)-1β inflammation pathway plays a main role in resorption of tooth root and bone; Al-Qawasmi et al. examined genetic factors predisposing to external apical root resorption (20). IL-1β is a pro-inflammatory cytokine that is involved in inflammatory responses. They suggested that polymorphisms in IL-1 gene (presence of IL-1β + C3953 allele and IL-1RN + 2018C allele) are involved in root resorption pathogenesis. Urban et al. demonstrated that IL-1 gene polymorphism presents a significantly higher risk for development of pathological root resorptions (21). Linares et al. also suggested that variations in the interleukin 1 receptor antagonist gene (rs419598) _and not only in the IL-1β gene (rs1800587) _are determinants of a predisposition to post-orthodontic external apical root resorption. Activation of IL-1β inflammation pathway due to chronic pulpal inflammation might be responsible for external root resorption in the presented case, which should be studied more in future (22).

## 4. Conclusion

Although the exact etiology of the idiopathic root resorption is still unknown, the present case showed that there might be relationship between extensive pulpal inflammation and the idiopathic resorption. Further studies on the molecular basis of the root resorption are recommended.
